# Identification of Termite Species and Subspecies of the Genus *Zootermopsis* Using Near-Infrared Reflectance Spectroscopy

**DOI:** 10.1673/031.007.1801

**Published:** 2007-04-05

**Authors:** Benjamin T. Aldrich, Elizabeth B. Maghirang, Floyd E. Dowell, Srinivas Kambhampati

**Affiliations:** ^1^ Department of Entomology, Kansas State University, Manhattan, KS 66506 USA; ^2^ Department of Anesthesia, University of Iowa, Iowa City, IA 52242 USA (Present Address); ^3^ Engineering Research Unit, USDA, ARS, GMPRC, Manhattan, KS 66502 USA

**Keywords:** species identification, neural network, *Zootermopsis angusticollis*, *Zootermopsis laticeps*, *Zootermopsis nevadensis*, *Z*. *n. nuttingi*, *Z. n. nevadensis*

## Abstract

Dampwood termites of the genus *Zootermopsis* (Isoptera: Termopsidae) are an abundant group of basal termites found in temperate forests of western North America. Three species are currently recognized in the genus and one of these species is subdivided into two subspecies. Although morphological and genetic characters are useful in differentiating among the three species and the two subspecies, respectively, only hydrocarbon analysis can enable differentiation both among the three species and the two subspecies. Due to the limitations of hydrocarbon analysis, such as the need for fresh specimens, alternative methods that could rapidly and accurately identify *Zootermopsis* would be useful. Using a partial least squares analysis of near-infrared spectra, each of the *Zootermopsis* species and subspecies were identified with greater than 95% and 80% accuracy, respectively. Neural network analysis of the near-infrared spectra successfully enabled the identification of the species and subspecies with greater than 99% accuracy. The inexpensive, reproducible, and rapid nature of near-infrared spectroscopy makes it a viable alternative to morphological, hydrocarbon, or genetic analysis for identifying *Zootermopsis.*

## Introduction

Dampwood termites of the genus *Zootermopsis* are the only endemic termites in Nearctic temperate forests of western North America (Eggleton 2000). Morphological characteristics have been used to recognize three species in the genus: *Zootermopsis nevadensis* (Hagen), *Zootermopsis angusticollis* (Hagen), and *Zootermopsis laticeps* (Banks). The shape of the pronotum and the size of the alates distinguish *Z. laticeps* from *Z. angusticollis* and *Z. nevadensis* (Sumner 1933; Nutting 1965). *Zootermopsis angusticollis* can be distinguished from *Z. nevadensis* by body and wing color and wing size (Sumner 1933; Weesner 1965). More recently, Thorne and Haverty (1989) identified differences in the shape of worker mandibles among all three species.

Haverty et al. 1988 used hydrocarbon analysis to identify *Zootermopsis* species and found four unique cuticular hydrocarbon phenotypes instead of three. Phenotype IV was specific to *Z. laticeps*, phenotype II was specific to *Z. angusticollis*, and phenotypes I and III were observed in *Z*. *nevadensis.* Assuming cuticular hydrocarbons are species specific (Howard and Blomquist 1982), Haverty et al. 1988 concluded that at least one more species of *Zootermopsis* might exist. This study was followed by an investigation into agonistic behavior among the various cuticular hydrocarbon phenotypes (Haverty and Thorne 1989). Agonistic behavior was pronounced among the three species as well as between phenotypes I and III (both *Z*. *nevadensis*). Furthermore, *Z*. *nevadensis* hydrocarbon phenotypes I and III are largely allopatric in distribution (Haverty and Thorne 1989; Thorne et al. 1993). Using agonistic behavior, allopatric distribution, and cuticular hydrocarbons as characteristics, Haverty and Thorne (1989) elevated the two hydrocarbon phenotypes of *Z. nevadensis* to subspecies status and named them *Z. n. nevadensis* (phenotype I) and *Z. n. nuttingi* (phenotype III).

Much is known about the biology of *Zootermopsis* (reviewed in Krishna and Weesner 1969; Thorne 1997; Abe et al. 2000; Thorne and Traniello 2003). In addition, considerable information concerning the distribution, biogeography, population genetics, and phenotypic relationships (cuticular hydrocarbons) has been accumulated for *Zootermopsis* (Haverty et al. 1988; Thorne et al. 1993; Aldrich and Kambhampati unpublished data). However, methods for the identification of *Zootermopsis* species and subspecies, other than hydrocarbon analysis, are currently lacking. Using allozymes, Korman et al. 1991 found fixed allelic differences among *Zootermopsis* species but not for the subspecies. Aldrich and Kambhampati (unpublished data) found subspecies specific genetic differences in the mitochondrial cytochrome oxidase subunit I gene, but differences in this gene among the *Zootermopsis* species are yet to be investigated.

Although hydrocarbon analysis can successfully identify *Zootermopsis*, a rapid, easy, and reliable technique would be useful. Recent studies have demonstrated the utility of near-infrared reflectance spectroscopy for the identification of various insect species (Dowell et al. 1999; Cole et al. 2003). Cuticular hydrocarbon differences among the *Zootermopsis* species and subspecies (Haverty and Thorne 1989) increases the likelihood that each *Zootermopsis* taxon will show a unique near-infrared reflectance spectrum. As discussed by Cole et al. 2003, this system has advantages over other identification techniques (morphological, genetic, and hydrocarbons) due to the relative speed of sample analysis and the inexpensive nature of the technology. Additionally, specimens preserved in alcohol can be utilized in near-infrared reflectance spectroscopy, whereas cuticular hydrocarbon analysis requires fresh specimens. Therefore, this study was undertaken with the goal of using near-infrared reflectance spectroscopy to discriminate among all species and subspecies of *Zootermopsis.*

## Materials and Methods

### Termite and spectral data collection

Specimens of *Z. angusticollis* and *Z*. *laticeps* were obtained from Dr. Michael Haverty (USDA Forest Service, Berkeley, CA). *Zootermopsis nevadensis* subspecies (Z. *n. nuttingi* and *Z*. *n. nevadensis*) were collected in California during June, 2003 by BTA and SK. The *Z*. *angusticollis* and *Z*. *laticeps* species were identified using cuticular hydrocarbons (Haverty et al. 1988) and the *Z*. *nevadensis* subspecies were identified using subspecies-specific mitochondrial cytochrome oxidase subunit I gene haplotypes (Aldrich and Kambhampati unpublished data). All specimens used in this study were stored in 95% ethanol prior to near-infrared reflectance spectroscopy. To account for any intra-species and subspecies variability in chemical composition, four to nine different colonies of each species and subspecies were included in this analysis (see Table 1). Only the larger workers were analyzed to maintain consistency in caste and size among the samples. The number of individuals included in the analysis for each species/subspecies was: 50 for Z. *angusticollis*, and 56 for *Z. laticeps*, 170 for *Z*. *n*. *nuttingi*, and 170 for *Z*. *n*. *nevadensis*.

**Table 1.**
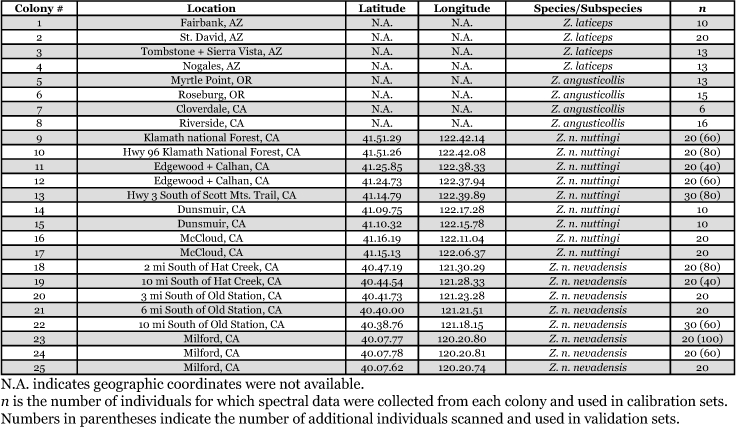
Sampling data for *Zootermopsis* species and subspecies.

Prior to spectral data collection, specimens were removed from ethanol and air-dried for 30 minutes under ambient room conditions. Specimens were positioned ventral side-up in a V-shaped black plastic sample trough (12mm long × 10mm wide × 5mm deep). The sample trough was illuminated with white light from a fiber bundle positioned 13 mm from the top of the trough and oriented at a 45° angle with respect to the trough. Visible and near-infrared reflectance (400–1700 nm) energy was carried to a Perten DA 7000 diode-array spectrometer (Perten Industries, Springfield, IL, USA) via a reflectance probe (2-mm in diameter) oriented vertically 9.5 mm from the top of the trough. Near-infrared reflectance spectra were recorded as absorbance or log(1/reflectance) at 5 nm intervals in the 400 to 1700 nm range of the spectrum. Background spectrum, measured using the empty trough just prior to data collection, was subtracted from all spectra. For each insect, the spectrophotometer automatically collected 15 spectra and yielded an averaged spectrum. Collecting and averaging the 15 spectra took less than 1 second per insect and preparing, positioning, and collecting the spectral data for all 446 samples took less than 4 hours.

### Data analysis

Spectral data were analyzed using either partial least squares regression (Martens and Naes 1989) using GRAMS ver. 7 software (Thermo Galactic, Salem, NH, USA) for two-way comparisons, or using a neural network (Neuroshell Classifier 2.01, Frederick, MD, USA) when comparing all four *Zootermopsis* taxa. The two-way comparisons were made using partial least squares regression by assigning a value of 1 or 2 for each pairwise combination of *Z*. *laticeps*, *Z*. *angusticollis*, *Z*. *n. nevadensis*, and *Z*. *n. nuttingi* (e.g., *Z. laticeps* = 1, *Z. angusticollis* = 2) to create calibrations based on the corresponding near-infrared reflectance spectra or independent variable. Validation sets were created by removing 10% of the individuals randomly from each species/subspecies. Randomly sampled individuals were treated as unknowns and subjected to a rejection threshold value of 1.5 (i.e., midpoint between the assigned values of 1 and 2 for the pairwise comparison). Thus, any individuals assigned a value of 1 or 2 in partial least squares regression that were classified above or below 1.5, respectively, would be considered misclassifications. In a second test of the calibration model, a validation set of 660 *Z. nevadensis* specimens (320 *Z. n. nuttingi* and 340 *Z*. *n*. *nevadensis*) was scanned, classified using the calibration created above, and subjected to a rejection threshold value of 1.5. To determine if we could accurately identify termite samples that were independent of the calibration set (i.e. a test set) a resampling procedure was done in which the spectral data for one *Z*. *n*. *nevadensis* and one *Z*. *n*. *nuttingi* colony was randomly removed. The remaining colonies were used to create calibrations and the randomly removed test set colonies were classified as described above for the validation set. The test set experiments were limited to the two *Z*. *nevadensis* subspecies. The wavelengths important in classifying *Zootermopsis* were determined based on partial least squares regression coefficients and differences in spectra. Accuracy of identification was determined using percent correct classification, coefficient of determination (R^2^), and standard error of cross validation (SECV).

**Table 2.**
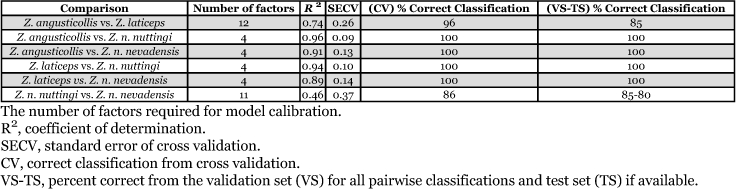
Statistical measures for PLS analysis among *Zootermopsis* species and subspecies.

Classifications for all four *Zootermopsis* taxa in a single test were obtained by analyzing spectral data using a neural network classification program (Neuroshell Classifier 2.01, Frederick, MD). In brief, the program was provided near-infrared reflectance spectral data (input nodes) and the corresponding subspecies type for each spectra (output nodes). The neural network then used a learning algorithm to create a classification scheme or calibration model based on the data provided. During training, the neural network minimizes the number of hidden neurons (steps connecting input nodes to output nodes) to reach an optimal calibration model. For the neural network analyses, 10% of the individuals were removed randomly from each species/subspecies group, treated as a validation set, and used for testing calibration models. The remaining 90% of samples were used as training (calibration) sets. The calibration and validation data sets consisted of 125 input nodes corresponding to the absorbance values at 10 nm increments ranging from 450–1700 nm and four outputs (*Z*. *laticeps*, *Z*. *angusticollis*, *Z*. *n*. *nevadensis*, and *Z*. *n*. *nuttingi*). The calibration data set contained 400 rows of data (individual spectral data) and the validation data set contained 46 rows of data. Neural network calibration models were used to determine which input nodes contributed to classifications.

**Table 3.**
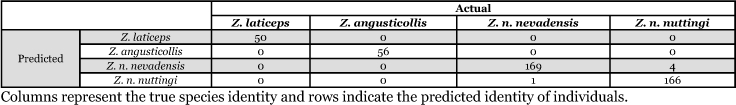
Accuracy of identification during training of neural network.

## Results

### Classification information

*Zootermopsis* specimens were sampled from 25 different colonies in Arizona, California, and Oregon. The geographic location of each colony and the number of individuals for which spectral data were collected for each colony are listed in Table 1. Selected statistical measures (percent correct classification, R^2^, and SECV) from partial least squares analyses and the number of factors used for calibration are listed in Table 2. Cross validation data showed that the average percent correct classification for all pairwise comparisons was 97% ± 2.36 (S.E.) and ranged from 86% to 100%. Calibration models had an average R^2^ value of 0.82 ± 0.08 (S.E.) with values ranging from 0.46 to 0.96. The lowest percent correct classification was for the comparison between *Z*. *n*. *nuttingi* and *Z*. *n*. *nevadensis* (86%) followed by the *Z*. *laticeps* and *Z*. *angusticollis* comparison (96%). The remaining comparisons resulted in 100% correct classification. The *Zootermopsis* species and subspecies were correctly identified with an average accuracy of 95% ± 3.14 (S.E.) and a minimum accuracy of 85% in validation sets (Table 2). Increasing the number of individuals in the validation set for classification of *Z*. *nevadensis* subspecies (i.e. 10% vs. 660 individuals) lowered the accuracy of prediction only slightly to 84%. Finally, the test set experiments were able to correctly classify the subspecies with an average accuracy of 80% ± 2.85.

**Figure 1.**
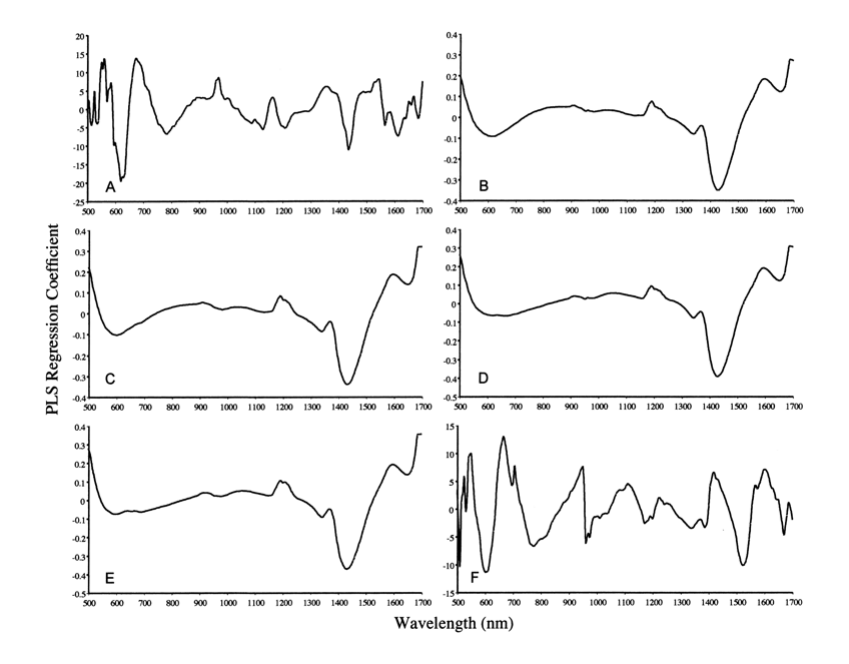
Partial least squares regression coefficients used for indicating important visible and near-infrared reflectance wavelengths for identification of *Zootermopsis* species and subspecies. The comparison tested in each graph and numbers of factors needed for each comparison are as follows: (A.) = *Z. angusticollis* versus *Z*. *laticeps* (12 factors), (B.) = *Z. angusticollis* versus *Z. n. nuttingi* (4 factors), (C.) = *Z.* *angusticollis* versus *Z*. *n*. *nevadensis* (4 factors), (D.) = *Z*. *laticeps* versus *Z*. *n*. *nuttingi* (4 factors), (E.) = *Z*. *laticeps* versus *Z*. *n*. *nevadensis* (4 factors), (F.) = *Z*. *n*. *nuttingi versus Z. n. nevadensis* (11 factors).

Neural network analyses classified *Zootermopsis* species with 99% accuracy in training sets (Table 3). The remaining 1% was due to misclassifications between *Z*. *n. nevadensis* and *Z*. *n. nuttingi.* Training of the neural network was completed in 1 minute and 5 seconds and required an optimal number of 46 hidden neurons. Classification of unknown individuals in the validation set took less than five seconds and the network was able to correctly identify unknowns with 100% accuracy.

### Chemical differences contributing to classification

Important wavelengths for identifying *Zootermopsis* can be derived from partial least squares correlation coefficients outputs shown in Figure 1. The wavelengths could be divided into those important in all pairwise classifications, in two or more classifications, or in a single classification. Wavelengths useful in identifying *Zootermopsis* in all six pairwise classifications occurred at approximately 1195, 1330, 1360, 1570, 1670, and 1695 nm. Wavelengths important in two or more classifications occurred at approximately 610, 680, 790, 950, 1130, 1415, 1440, 1520, 1620, and 1685 nm. Finally, wavelengths utilized in only a single classification occurred at about 1100, 1170, 1215, 1395, and 1540 nm. More than half of the important wavelength regions (1130, 1195, 1215, 1330, 1360, 1395, 1415, 1440, 1620, 1670, and 1695 nm) correspond to CH_2_ and CH_3_ first, second, and combination overtones (Shenk et al. 1992). Other characteristics important in identifying *Zootermopsis* include color differences (610 and 680 nm), HC=CH C-H second overtone (1170 nm), CONH N-H stretch first overtone (1570 nm), and aromatic C-H stretch first overtone (1685 nm). There was some similarity in the partial least squares regression profiles for some pairwise comparisons (Figure 1: B, C, D, and E) suggesting that the same wavelengths or chemical functional groups are important in differentiating these four pairs of samples. Most of the wavelengths important in partial least squares classifications were also the most important factors in neural network classifications (data not shown). Only the wavelengths at 950, 1170, 1685, and 1695 nm were useful in partial least squares classifications but not neural network classifications.

## Discussion

Near-infrared reflectance spectroscopy enabled the identification of *Zootermopsis* species and subspecies with an average accuracy of 95% using partial least squares analysis and 100% using neural network analysis. Although both partial least squares and neural networks were successful in identifying *Zootermopsis* with a high degree of accuracy, the neural network is the preferred method for two reasons. First, the neural network yielded a higher percentage of correct classifications in validation sets compared to partial least square. Second, partial least squares is able to perform only two-way classifications whereas the neural network is able to classify all taxa simultaneously in a single test. Thus, the former method is useful only if the unknown sample is narrowed down to two alternative taxa whereas the latter method requires no prior knowledge of the sample type.

Given that *Zootermopsis* species and subspecies can be identified by differences in cuticular hydrocarbons, it is not surprising that these termites can also be differentiated using near-infrared reflectance spectroscopy. Partial least squares regression coefficients indicated that a majority of the wavelengths useful in identifying *Zootermopsis* corresponded to CH_3_ and CH_2_ first, second, and combination C-H bond vibrations (Shenk et al. 1992). CH_3_ and CH_2_ are common structural features of compounds that make up insect epicuticular lipids, including hydrocarbons (Lockey 1988). Using near-infrared reflectance spectroscopy, Dowell et al. 1999 found that differences in cuticular lipids with peaks at 1130 and 1670 nm are important in identifying several species of stored grain insects. Regression coefficients indicated that the 1130 and 1670 nm wavelengths are also important for discriminating among the *Zootermopsis* species. Thus, each *Zootermopsis* species and subspecies may have unique mixtures of cuticular lipids, which are reflected by differences in near-infrared reflectance spectra. Furthermore, near-infrared reflectance spectroscopy may be detecting many of the same differences in cuticular hydrocarbons reported by Haverty and Thorne (1989). However, additional evidence is needed to identify the true nature of the species-specific molecules and confirm the above predictions.

In summary, our results show that near-infrared reflectance spectra analyzed by partial least squares or neural network spectral analysis can be used to rapidly and accurately identify *Zootermopsis* species and subspecies. In addition to *Zootermopsis*, this technique could prove useful in identification of other termite species. General advantages of this technique include the ability to utilize ethanol-preserved specimens, rapid data collection and analysis, inexpensive equipment and operation, ease of use, and ability to identify species using the worker caste. Workers are typically the most abundant caste in termite colonies making near-infrared reflectance spectroscopy a useful identification technique. Assuming chemical differences between species and subspecies are maintained in soldiers and reproductives, near-infrared reflectance spectroscopy should prove useful in identifications using other castes as well. Near-infrared reflectance systems to repeat this work would cost about $20,000 U.S. Furthermore, portable near-infrared reflectance spectrometers designed for field use are currently available and can be purchased for around $65,000 U.S. Thus, researchers and termite control companies may find near-infrared reflectance systems useful for rapid, on-site termite species identification.
